# Supplementary material for: Pyruvate Dehydrogenase Kinase 1 inhibition mediated oxidative phosphorylation enhancement in cartilage promotes osteoarthritis progression
Source: BMC Musculoskelet Disord. 2023 Jul 20;24:597. doi: 10.1186/s12891-023-06585-6 (PMC10357736; doi:10.1186/s12891-023-06585-6)
Supplement: Supplementary file 1 — Additional file 1. [file 12891_2023_6585_MOESM1_ESM.zip › KEGG.Gsea.1653622432464/KEGG_ABC_TRANSPORTERS.html]

Details for gene set KEGG\_ABC\_TRANSPORTERS[GSEA]

|  || Dataset | 55457\_collapsed\_to\_symbols.cls.cls #OA\_versus\_normal.cls.cls #OA\_versus\_normal\_repos |
| Phenotype | cls.cls#OA\_versus\_normal\_repos |
| Upregulated in class | normal |
| GeneSet | KEGG\_ABC\_TRANSPORTERS |
| Enrichment Score (ES) | -0.21840738 |
| Normalized Enrichment Score (NES) | -0.7703047 |
| Nominal p-value | 0.8462867 |
| FDR q-value | 0.9712015 |
| FWER p-Value | 1.0 |
Table: GSEA Results Summary

  

Fig 1: Enrichment plot: KEGG\_ABC\_TRANSPORTERS      
 Profile of the Running ES Score & Positions of GeneSet Members on the Rank Ordered List

  

| SYMBOL | TITLE | RANK IN GENE LIST | RANK METRIC SCORE | RUNNING ES | CORE ENRICHMENT || 1 | ABCC6 | ATP binding cassette subfamily C member 6 [Source:HGNC Symbol;Acc:HGNC:57] | 729 | 0.587 | 0.0130 | No |
| 2 | ABCG1 | ATP binding cassette subfamily G member 1 [Source:HGNC Symbol;Acc:HGNC:73] | 1711 | 0.396 | -0.0163 | No |
| 3 | ABCB6 | ATP binding cassette subfamily B member 6 (Langereis blood group) [Source:HGNC Symbol;Acc:HGNC:47] | 2717 | 0.278 | -0.0614 | No |
| 4 | ABCC8 | ATP binding cassette subfamily C member 8 [Source:HGNC Symbol;Acc:HGNC:59] | 2844 | 0.266 | -0.0397 | No |
| 5 | ABCG5 | ATP binding cassette subfamily G member 5 [Source:HGNC Symbol;Acc:HGNC:13886] | 2942 | 0.256 | -0.0168 | No |
| 6 | ABCD2 | ATP binding cassette subfamily D member 2 [Source:HGNC Symbol;Acc:HGNC:66] | 3031 | 0.246 | 0.0056 | No |
| 7 | ABCC4 | ATP binding cassette subfamily C member 4 [Source:HGNC Symbol;Acc:HGNC:55] | 3344 | 0.217 | 0.0071 | No |
| 8 | ABCA3 | ATP binding cassette subfamily A member 3 [Source:HGNC Symbol;Acc:HGNC:33] | 3549 | 0.198 | 0.0147 | No |
| 9 | ABCC3 | ATP binding cassette subfamily C member 3 [Source:HGNC Symbol;Acc:HGNC:54] | 4200 | 0.138 | -0.0195 | No |
| 10 | ABCA12 | ATP binding cassette subfamily A member 12 [Source:HGNC Symbol;Acc:HGNC:14637] | 4451 | 0.117 | -0.0250 | No |
| 11 | ABCA8 | ATP binding cassette subfamily A member 8 [Source:HGNC Symbol;Acc:HGNC:38] | 4518 | 0.112 | -0.0169 | No |
| 12 | ABCB7 | ATP binding cassette subfamily B member 7 [Source:HGNC Symbol;Acc:HGNC:48] | 4669 | 0.099 | -0.0168 | No |
| 13 | ABCC2 | ATP binding cassette subfamily C member 2 [Source:HGNC Symbol;Acc:HGNC:53] | 5206 | 0.056 | -0.0519 | No |
| 14 | ABCA2 | ATP binding cassette subfamily A member 2 [Source:HGNC Symbol;Acc:HGNC:32] | 5349 | 0.044 | -0.0577 | No |
| 15 | ABCB9 | ATP binding cassette subfamily B member 9 [Source:HGNC Symbol;Acc:HGNC:50] | 6442 | -0.038 | -0.1380 | No |
| 16 | ABCG4 | ATP binding cassette subfamily G member 4 [Source:HGNC Symbol;Acc:HGNC:13884] | 6574 | -0.047 | -0.1426 | No |
| 17 | TAP1 | "transporter 1, ATP binding cassette subfamily B member [Source:HGNC Symbol;Acc:HGNC:43]" | 6633 | -0.052 | -0.1409 | No |
| 18 | ABCB8 | ATP binding cassette subfamily B member 8 [Source:HGNC Symbol;Acc:HGNC:49] | 7303 | -0.102 | -0.1808 | No |
| 19 | ABCG2 | ATP binding cassette subfamily G member 2 (Junior blood group) [Source:HGNC Symbol;Acc:HGNC:74] | 7473 | -0.116 | -0.1801 | No |
| 20 | ABCA6 | ATP binding cassette subfamily A member 6 [Source:HGNC Symbol;Acc:HGNC:36] | 7655 | -0.131 | -0.1786 | No |
| 21 | ABCB11 | ATP binding cassette subfamily B member 11 [Source:HGNC Symbol;Acc:HGNC:42] | 7825 | -0.143 | -0.1748 | No |
| 22 | ABCB1 | ATP binding cassette subfamily B member 1 [Source:HGNC Symbol;Acc:HGNC:40] | 8228 | -0.173 | -0.1855 | No |
| 23 | ABCD4 | ATP binding cassette subfamily D member 4 [Source:HGNC Symbol;Acc:HGNC:68] | 8652 | -0.205 | -0.1941 | Yes |
| 24 | ABCA7 | ATP binding cassette subfamily A member 7 [Source:HGNC Symbol;Acc:HGNC:37] | 8856 | -0.222 | -0.1836 | Yes |
| 25 | ABCD3 | ATP binding cassette subfamily D member 3 [Source:HGNC Symbol;Acc:HGNC:67] | 8877 | -0.223 | -0.1588 | Yes |
| 26 | ABCD1 | ATP binding cassette subfamily D member 1 [Source:HGNC Symbol;Acc:HGNC:61] | 8930 | -0.226 | -0.1359 | Yes |
| 27 | ABCC5 | ATP binding cassette subfamily C member 5 [Source:HGNC Symbol;Acc:HGNC:56] | 9066 | -0.237 | -0.1183 | Yes |
| 28 | ABCC10 | ATP binding cassette subfamily C member 10 [Source:HGNC Symbol;Acc:HGNC:52] | 9218 | -0.250 | -0.1004 | Yes |
| 29 | ABCC9 | ATP binding cassette subfamily C member 9 [Source:HGNC Symbol;Acc:HGNC:60] | 9346 | -0.259 | -0.0796 | Yes |
| 30 | ABCB4 | ATP binding cassette subfamily B member 4 [Source:HGNC Symbol;Acc:HGNC:45] | 9807 | -0.302 | -0.0795 | Yes |
| 31 | ABCA1 | ATP binding cassette subfamily A member 1 [Source:HGNC Symbol;Acc:HGNC:29] | 9874 | -0.309 | -0.0479 | Yes |
| 32 | ABCA4 | ATP binding cassette subfamily A member 4 [Source:HGNC Symbol;Acc:HGNC:34] | 10554 | -0.379 | -0.0558 | Yes |
| 33 | CFTR | CF transmembrane conductance regulator [Source:HGNC Symbol;Acc:HGNC:1884] | 10920 | -0.420 | -0.0343 | Yes |
| 34 | ABCC1 | ATP binding cassette subfamily C member 1 [Source:HGNC Symbol;Acc:HGNC:51] | 11462 | -0.497 | -0.0174 | Yes |
| 35 | TAP2 | "transporter 2, ATP binding cassette subfamily B member [Source:HGNC Symbol;Acc:HGNC:44]" | 11616 | -0.519 | 0.0323 | Yes |
| 36 | ABCA5 | ATP binding cassette subfamily A member 5 [Source:HGNC Symbol;Acc:HGNC:35] | 11884 | -0.568 | 0.0790 | Yes |
Table: GSEA details [plain text format]

  

Fig 2: KEGG\_ABC\_TRANSPORTERS      
 Blue-Pink O' Gram in the Space of the Analyzed GeneSet

  

Fig 3: KEGG\_ABC\_TRANSPORTERS: Random ES distribution      
 Gene set null distribution of ES for **KEGG\_ABC\_TRANSPORTERS**
